# Genetic variation of *Cerastium alpinum* L. from Babia Góra, a critically endangered species in Poland

**DOI:** 10.1007/s13353-022-00731-x

**Published:** 2022-11-02

**Authors:** Sylwia Eryka Milarska, Piotr Androsiuk, Piotr Tomasz Bednarek, Keith Larson, Irena Giełwanowska

**Affiliations:** 1 Department of Plant Physiology, Genetics and Biotechnology, Faculty of Biology and Biotechnology, University of Warmia and Mazury in Olsztyn, ul. M. Oczapowskiego 1A, 10-719 Olsztyn, Poland; 2 Plant Breeding and Acclimatization Institute – National Research Institute, Radzików, 05-870 Błonie, Poland; 3 Climate Impacts Research Centre, Department of Ecology and Environmental Sciences, Umeå University, 901 87 Umeå, Sweden

**Keywords:** Arctic-alpine perennial, Population differentiation, Genetic structure, Retrotransposon-based iPBS markers, Species conservation

## Abstract

Babia Góra massif is the only site of occurrence of the *Cerastium alpinum* L. in Poland, an arctic-alpine perennial plant with a wide distribution in North America, northwestern Asia, and Europe. To determine whether the isolated Polish populations are genetically distinct, we have performed an evaluation of *C. alpinum* from Babia Góra with the use of iPBS markers. A total number of 133 individuals of *C. alpinum* from seven populations representing four localizations of the species were analyzed, i.e., from Babia Góra (Poland), Alps (Switzerland), Nuolja massif (Sweden), and Kaffiøyra (Svalbard, Norway). Genetic analysis of all *C. alpinum* samples using eight PBS primers identified 262 bands, 79.4% of which were polymorphic. iPBS markers revealed low genetic diversity (average *H*_*e*_ = 0.085) and high population differentiation (*F*_*ST*_ = 0.617)*.* AMOVA results confirmed that the majority of the genetic variation (62%) was recorded among populations. The grouping revealed by PCoA showed that *C. alpinum* from Svalbard is the most diverged population, *C. alpinum* from Switzerland and Sweden form a pair of similar populations, whereas *C. alpinum* from the Babia Góra form a heterogeneous group of four populations. Results of isolation by distance analysis suggested that the spatial distance is the most probable cause of the observed differentiation among populations. Although significant traces of a bottleneck effect were noted for all populations of *C. alpinum* from Babia Góra, the populations still maintain a low but significant level of genetic polymorphism. These results are of great importance for developing conservation strategies for this species in Poland.

**Supplementary Information:**

The online version contains supplementary material available at 10.1007/s13353-022-00731-x.

## Introduction

The genus *Cerastium* (Caryophyllaceae) consists of over 200 species (Plants of the World Online, http://www.plantsoftheworldonline.org), the majority of which are spread in the Northern Hemisphere in the temperate climate zone, but only a few of them are cosmopolitan species. *Cerastium alpinum* is a perennial that grows in rocky, sandy, or gravely substrates and is characterized by its matted growth form, hairy basal leaves, flowering stems, white, five-petalled flowers about 20 mm in diameter, and a branched root system (Porsild and Cody 1980; Clapham et al. 1987; Rønning 1999). Flowering is initiated relatively late, from June to August. The species is generally cross-pollinated by insects, although self-pollination occurs (Totland and Schulte-Herbrüggen 2003; Parusel 2008). Seeds are dispersed by gravity—they are sown mainly within a close vicinity of the source plant (Parusel 2008). In Poland, its only natural sites are located in the Babia Góra massif (Żywiec Beskids, Western Carpathians) within the area of the Babia Góra National Park (Parusel 2001). *C. alpinum* can be found on high mountain grasslands where it forms one-species aggregations on rock shelves, in rock crevices, and on stony debris (Parusel 2014). Most of these sites were recorded in the subalpine zone, from 1480 to 1680 m.a.s.l. (Parusel 2001). Due to the limited area of occurrence and spatial isolation of its sites (the closest populations are in the Alps and the Eastern Carpathians; Parusel 2001), *C. alpinum* was included in the Polish Red Data Book of Plants and received the status of a critically endangered species in Poland (Parusel 1993, 2014; Mirek and Piękoś-Mirkowa 2008). This makes it a critical component of Polish flora that requires special attention, i.e., increased interdisciplinary activity to develop effective strategies for species conservation and restoration.

The subject of conservation genetic studies is to understand the dynamics of genes in populations threatened by extinction, to apply the techniques and concepts of genetics, and to use them to address problems in conservation biology (Hedrick and Miller 1992). To plan an effective conservation strategy, it is necessary to know the current genetic condition of the population. Genetic diversity is influenced by population size, gene flow, the mating system, and other facultative factors like genetic drift, founder effects or bottleneck, and subsequent inbreeding (Ellstrand and Elam 1993). Inbreeding depression, consequently, can lead to the accumulation of deleterious mutations, a reduction in plant fitness, and a further reduction in population size (Ellstrand and Elam 1993; Lynch et al. 1995; Young et al. 1996). Another factor leading to genetic degradation is spatial isolation, which may restrict or prevent gene flow. Due to being rare and in a remote area, *C. alpinum* from Babia Góra may be exposed to the above problems, which could make the risk of local extinction higher.

In recent decades, various PCR-based techniques such as randomly amplified polymorphic DNA (RAPD; Williams et al. 1990), inter simple sequence repeat (ISSR; Zietkiewicz et al. 1994), amplified fragment length polymorphism (AFLP; Vos et al. 1995), or simple sequence repeats (SSR; Tautz 1989) have been widely used for assessment of the genetic diversity of many plants (e.g., Arif et al. 2010; Amiteye 2021) and animal (e.g., Arif and Khan 2009; Yang et al. 2013) species. Knowledge of the level of genetic polymorphism maintained in a particular population and information about the relatedness between individuals is essential for establishing efficient conservation management programs.

Unfortunately, the knowledge of the genetic diversity of *C*. *alpinum* and the whole genus *Cerastium* is minimal. The genetic studies for the genus *Cerastium* include the RAPD and SCAR markers for *C*. *arcticum* (Hagen et al. 2001) or AFLP technique for *C*. *dinaricum* (Kutnjak et al. 2014), *C*. *decalvans* (Niketić et al. 2022), *C*. *sylvaticum*, *C*. *subtriflorum* (Skubic et al. 2018), *C*. *hekuravense* (Caković et al. 2018) and *C*. *grandiflorum*, *C*. *decalvans* and *C*. *dinaricum* (Đurović et al. 2021). Most molecular studies of *C. alpinum* involved populations found in Svalbard, Greenland, Iceland, Norway, Sweden, and Finland and relied on analyses of isoenzymatic polymorphism (Nyberg Berglund et al. 2006). Nonetheless, they concentrated on the issue of hybridization and introgression between *C. alpinum* and other closely related species (i.e., *C. arcticum*, *C. nigrescens*) (Brysting and Borgen 2000; Hagen et al. 2002), as well as the identification and comparison of various isoenzymatic phenotypes from different populations to describe their geographic distribution and infer about the species’ history (colonization routes) in Fennoscandia. Scheen et al. (2004) also wrote about the phylogenetic relationships and biogeography of the genus *Cerastium*. Brysting (2000) and Brysting et al. (2011) researched how the number of chromosomes might vary in some *Cerastium* species.

The iPBS (inter primer binding sites) method described by Kalendar et al. (2010) is based on the virtually universal presence of a tRNA complement as a reverse transcriptase primer binding site (PBS) in long terminal repeat (LTR) retrotransposons. It does not require prior knowledge of the sequence, making it suitable for many objects, including “orphan crops” with underdeveloped marker systems. This marker system has been successfully used for the molecular characterization of different plant species, such as chickpea (Andeden et al. 2013), pea (Baloch et al. 2015), quinoa (Hossein-Pour et al. 2019), grapevine (Milovanov et al. 2019), lianas from genus *Gnetum* (Doungous et al. 2020), onion (Khapilina et al. 2021a, 2021b), or pathogenic fungi, e.g., representatives of genus *Alternaria* (Turzhanova et al. 2020) and *Rhizoctonia* (Erper et al. 2021). This method has also found application in phylogenetic research of saffron (Bayat et al. 2018) and emmer wheat (Arystanbekkyzy et al. 2019). Our previous studies demonstrated the usefulness of iPBS markers for genetic diversity studies in non-model plants, such as *Colobanthus quitensis* (Koc et al. 2018), *Poa annua* (Androsiuk et al. 2019), and *Deschampsia antarctica* (Androsiuk et al. 2020), for which knowledge on their genome sequence and organization is limited. The retrotransposon-based DNA marker system used in these studies effectively assessed DNA polymorphism between individuals and among populations.

The objectives of this study were as follows: (1) to test the suitability of iPBS to detect genetic polymorphism within and among *C*. *alpinum* populations; (2) to estimate genetic differentiation and relationships among studied *C*. *alpinum* populations from different geographic regions; and (3) to evaluate the genetic condition of *C*. *alpinum* from the Babia Góra massif to provide information essential for the development of effective conservation strategies for the genetic resources of *C*. *alpinum* in Babia Góra.

## Materials and methods

### Material

The research material consisted of 133 individuals of *Cerastium alpinum* representing seven sampling sites (from now on referred to as populations) from Poland, Switzerland, Sweden, and Svalbard (Norway) (Table 1). Plants from Switzerland and Sweden were obtained in a dried form. In the case of *C*. *alpinum* from Svalbard, the original material consisted of seeds collected during the Arctic expedition to Nicolaus Copernicus University Polar Station in Spitsbergen in 2012. Polish *C*. *alpinum* populations were collected in Babia Góra National Park after obtaining the permission of the Polish Ministry of Environment. All Polish sampling sites are scattered on the Babia Góra massif. To avoid damaging the *C. alpinum* individuals from these unique and valuable sampling sites, we collected only bags with mature seeds from 14 to 28 individuals from each localization; the sampled plants were ca. 10–20 m apart from each other to avoid collecting material representing the clonal genets as it is reported that *C*. *alpinum* can reproduce vegetatively (Rune 1957; Parusel 2008). Plants grown from seeds were maintained in the greenhouse of the Department of Plant Physiology, Genetics and Biotechnology, Faculty of Biology and Biotechnology at the University of Warmia and Mazury in Olsztyn, Poland. In the case of *C*. *alpinum* from Babia Góra massif, one seedling obtained from three to five germinated seeds originating from one seed bag, representing each sampled specimen, was selected for DNA extraction.

**Table 1:** The origin of *Cerastium alpinum* populations used in the study

Population	Sampling site	Coordinates (GPS)	Altitude (m.a.s.l.)	No. individuals
1	Poland Western Carpathians, Babia Góra National Park	49°34′28.54″ N; 19°32′13.95″ E	1592	23
2		49°34′27.04″ N; 19°32′4.64″ E	1620	14
3		49°34′27.21″ N; 19°31′57.80″ E	1618	28
4		49°34′24.14″ N; 19°31′47.27″ E	1629	16
5	Switzerland Graubünden, Samnaun-Gruppe, Piz Val Gronda	46°55′18″ N; 10°17′16″ E	2750	18
6	Svalbard (Norway) Svalbard archipelago, Spitsbergen, Kaffiøyra	78°40′34″ N; 11°49′38″ E	5	17
7	Sweden Norrbotten, Abisko National Park, Nuolja massif	68°22′04″ N; 18°44′2.60″ E	654–1164	17

The molecular analysis was performed using 133 *C*. *alpinum* individuals representing seven populations, ranging from 14 to 28 individuals per population. The size of a given population limited the number of sampled individuals.

### DNA extraction and iPBS genotyping

Genomic DNA from each individual was extracted with Genomic Mini AX Plant Spin kit (A&A Biotechnology). The amount and purity of isolated DNAs were estimated spectrophotometrically (NanoDrop). Additionally, the quality of DNA was verified on 1.5% (w/v) agarose staining with 0.5 µg/ml ethidium bromide.

Initially, 34 PBS primers (2074, 2076, 2079, 2080, 2085, 2217, 2220, 2221, 2224, 2228, 2229, 2231, 2232, 2237, 2238, 2240, 2241, 2242, 2249, 2251, 2253, 2272, 2277, 2373, 2374, 2376, 2378, 2381, 2389, 2391, 2393, 2395, 2399, 2415) were screened for *C. alpinum* (Kalendar et al. 2010), with the annealing temperature set at 56 °C for all primers. During this step, the quality of the band pattern was the factor that decided whether the particular PBS primer could be selected for further studies or not. To evaluate the quality of the band pattern for each tested PBS primer, we applied the scale of PCR efficiency described in the original paper by Kalendar et al. (2010). According to that scale, we could distinguish five types of iPBS band profiles: 0, no bands; 1, few and weak bands; 2, a few strong bands; 3, ≈10 strong bands; 4, many bands (good primer); 5, many strong and equally amplifying bands. Based on our observations for further steps of our study, we selected eight PBS primers with the highest score (Table S1). In the next step, we experimentally determined the optimal annealing temperature for selected primers, by performing PCR with the gradient of annealing temperature (50–60 °C). When the optimal annealing temperature for each selected PBS primer was determined (Table 2), the reproducibility of band profiles for each of selected PBS primer was verified. This verification was based on a comparison of the electrophoretic profiles for eleven individuals representing population 1 from Babia Góra, Poland. Data were generated and compared in two replicates. Gels were then checked to identify iPBS amplicons (bands) in one or both replicates.

**Table 2:** The sequence and specification of primers applied in the study

Primer	Sequence	Annealing temp. (°C)	Number of scored bands	Number of polymorphic bands
2217	5′-ACTTGGATGTCGATACCA-3′	56	35	24
2228	5′-CATTGGCTCTTGATACCA-3′	60	38	32
2229	5′-CGACCTGTTCTGATACCA-3′	60	26	17
2232	5′-AGAGAGGCTCGGATACCA-3′	58	34	30
2251	5′-GAACAGGCGATGATACCA-3′	60	34	31
2272	5′-GGCTCAGATGCCA-3′	56	30	17
2277	5′-GGCGATGATACCA-3′	56	34	34
2378	5′-GGTCCTCATCCA-3′	52	31	23
Total			262	208

Eight PBS primers that gave clearly identifiable and repeatable amplification products (bands) were selected for subsequent genetic diversity studies (Table 2). The iPBS amplification was performed according to the procedure described by Kalendar et al. (2010) with modifications (Androsiuk et al. 2015). Amplification products were analyzed by gel electrophoresis in 1.5% (w/v) agarose with 1 × TBE electrophoresis buffer at 100 V for 2 h and visualized with 0.5 μg/ml ethidium bromide.

### Data analysis

The amplified bands were scored as either 1 (present) or 0 (absent) across genotypes. Based on the obtained binary matrix of amplification products, the following genetic parameters were estimated with the use of GenAlEx 6.5 software (Peakall and Smouse 2006; 2012): the total number of bands per population (*N*_*B*_), percentage of polymorphic bands (*P*), Shannon’s information index (*I*), and expected heterozygosity (*H*_*e*_).

The genetic structure of the studied populations was inferred based on the Bayesian model-based clustering method implemented in Structure ver. 2.3.4 (Pritchard et al. 2000). The model probabilistically assigns individual multilocus genotypes to a user-defined number of clusters (*K*), achieving linkage equilibrium within clusters (Pritchard et al. 2000). We ran ten replicates with a burn-in of 100,000 iterations and with 500,000 iterations for Markov chain Monte Carlo for *K* ranging from 1 to 10 possible clusters. The analysis, with the implemented admixture model, was conducted without prior information on the original population of each sampled individual. The optimal number of clusters and ad hoc statistics Δ*K* (Evanno et al. 2005) was evaluated in Structure Harvester ver.0.6.94 (Earl and vonHoldt 2012). The genetic subdivision patterns of the analyzed *C. alpinum* populations were also investigated by principal coordinate analysis (PCoA) based on the matrix of Euclidean distances between individuals from all analyzed populations. This analysis was performed in PAST software (Hammer et al. 2001).

Analysis of molecular variance (AMOVA) was performed on Arlequin 3.5 software (Excoffier et al. 2005). For that analysis, the iPBS dataset was treated as haplotypic, consisting of a combination of alleles at one or several loci (Excoffier et al. 2005). The significance of the fixation indices was tested using a nonparametric permutation approach (Excoffier et al. 1992). The significance test was based on 1023 permutations.

The possible effect of increased spatial distance on the genetic structure of the studied populations of *C*. *alpinum* was also investigated. The spatial genetic structure was estimated by testing the significance of isolation by distance (IBD) using a Mantel test with 9999 permutations of the relationship between the matrix of pairwise *F*_*ST*_/(1 − *F*_*ST*_) and that of the logarithm of geographical distance between populations (Rousset 1997), using GenAlEx 6.5. The pairwise *F*_*ST*_ values were estimated in Arlequin 3.5 software, and their significance was also tested based on 110 permutations.

Tajima’s *D*, Fu’s *F*_*S*_ neutrality test, mismatch distribution, and demographic processes affecting populations were also performed using Arlequin 3.5 software (Excoffier et al. 2005).

Bottleneck ver. 1.2.02 (Cornuet and Luikart 1996) software was used to investigate the recent reduction of effective population size based on allele data frequencies for each *C. alpinum* population (Cornuet and Luikart 1996; Piry et al. 1999). This effect was studied using dominant markers in the infinite allele model (IAM) to test the mutation-drift hypothesis against the bottleneck hypothesis (Tero et al. 2003). The significance of potential bottleneck was estimated in the SIGN test, the standardized differences test, and the one-tailed Wilcoxon sign-rank test for heterozygosity excess with 10,000 simulations.

## Results

### Efficiency of applied PCR primers

Our analysis of *Cerastium alpinum* populations, using eight PBS primers, yielded 262 distinguished amplification products (bands) (Table 2, Table S2, File S1). The highest number of bands (38) was revealed by primer 2228, and the lowest number of bands (26) was obtained for primer 2229. On average, 32.75 bands were obtained per primer. Of the identified loci, 208 (79.39%) were polymorphic (Table 2).

Of a total of 262 scored bands, 20 (7.63%) were represented as private bands that were found only within one population and absent in the others (Table 3). The highest number of private bands (4) was revealed by primer 2228. The Svalbard population (6) appeared as the most abundant in private bands—six of them were scored in individuals representing that population, while for population 2, no private bands were found.

**Table 3:** Private alleles (bands) per locus and their respective frequencies revealed in studied *Cerastium alpinum* populations

Locus	Frequency	Population
2272_10	0.008	1
2228_11	0.105	3
2229_20	0.105	3
2232_23	0.030	3
2232_31	0.053	3
2272_21	0.015	3
2229_12	0.008	4
2251_21	0.120	4
2251_22	0.120	4
2217_10	0.053	5
2378_23	0.135	5
2228_12	0.120	6
2228_24	0.105	6
2229_16	0.120	6
2232_10	0.128	6
2272_18	0.015	6
2378_12	0.120	6
2217_4	0.060	7
2228_1	0.128	7
2378_26	0.008	7

### Genetic diversity and differentiation

The iPBS markers revealed the presence of genetic polymorphism between individuals within a population and genetic variation among populations (Table 4). The number of iPBS amplification products ranged from 172 for population 5 to 236 for population 3. The highest number of polymorphic bands was scored for population 3 (45.80%) and the lowest for population 5 (13.36%). On average (over loci and populations), 25.35% of iPBS loci were polymorphic. The genetic variation was assessed with two parameters: Shannon’s information index (*I*) and expected heterozygosity (*H*_*e*_). Population 3 had the highest values for both parameters, while population 5 had the lowest values.

**Table 4:** Population genetic characteristics for analyzed populations of *Cerastium alpinum*: number of bands (*N*_*B*_), percentage of polymorphic bands (*P*), Shannon’s information index (*I*), and expected heterozygosity (*H*_*e*_)

Population	*N*_*B*_	*P* (%)	*I*	*H*_*e*_
1	215	25.95	0.140	0.095
2	219	28.24	0.148	0.099
3	236	45.80	0.241	0.162
4	218	22.14	0.107	0.070
5	172	13.36	0.076	0.051
6	206	25.57	0.095	0.060
7	191	16.41	0.088	0.060
Mean over loci and populations	208.14	25.35	0.128	0.085

**Fig. 1:**
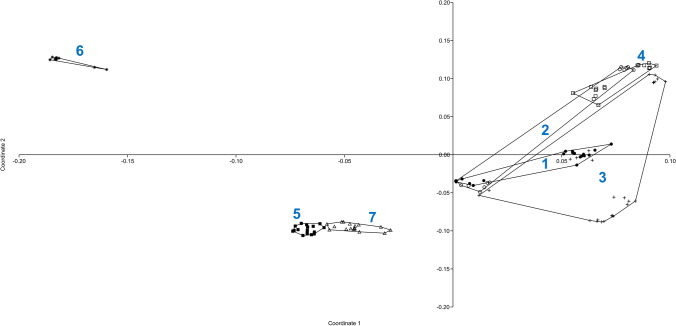
Plot of coordinate 1 versus coordinate 2 obtained by principal coordinates analysis (PCoA) based on Euclidean distances between all individuals from seven *Cerastium alpinum* populations. Populations are numbered according to Table 1: 1 (filled circle); 2 (open circle); 3 (plus sign); 4 (open square); 5 (filled square); 6 (asterisk); 7 (open triangle)

PCoA indicates that 51.56% of the variation was explained by the first three components (21.6, 18.83, and 11.13%, respectively). The projection of the analyzed populations on the first two axes is shown in Fig. 1. The grouping revealed by PCoA pointed to the high degree of similarity between populations 5 and 7. The Babia Góra populations (1–4) can be put together into one group, but population 6 is very different from the others along coordinates 1 and 2.

**Fig. 2:**
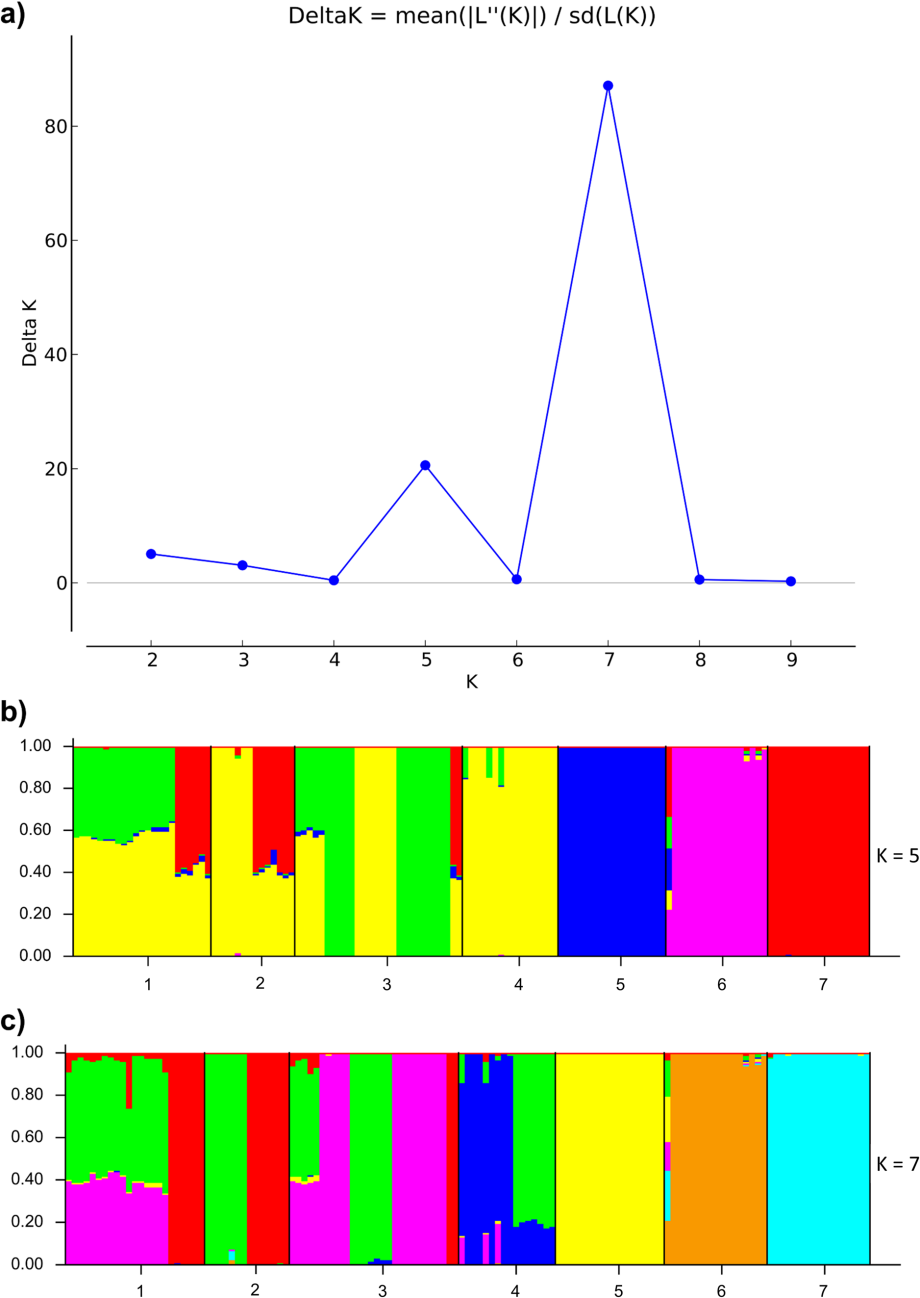
**a** The uppermost hierarchical level of genetic structure of *Cerastium alpinum* using STRUCTURE software (Pritchard et al. 2000); the optimal *K* value generated by Structure Harvester: **b** *K* = 5 and **c** *K* = 7

The Bayesian clustering analysis performed in Structure and Structure Harvester software revealed that Δ*K* has a maximum at *K* = 7, but a marked secondary peak at *K* = 5 was also found (Fig. 2a). Both populations’ structure bar plots generated by Structure software for *K* = 5 (Fig. 2b) and *K* = 7 (Fig. 2c) reveal a clear separation between populations 5, 6, and 7, which also appeared to be genetically homogenous. In the case of populations 1–4, a combination of diverse genotypes shared by all these populations can be observed, indicating admixture between these localizations. However, even then (for *K* = 7), in the case of populations 1 and 4, the predicted values of the proportion of membership of these populations to separate, individual clusters were > 0.7. The highest admixture was observed for populations 2 and 3, for which the predicted proportion of membership in the certain cluster was ≤ 0.499. For *K* = 5, an analogous pattern of population subdivision is observed. Pairwise *F*_*ST*_ analysis was also performed. The highest *F*_*ST*_ value (0.839) was observed between populations 5 and 6, whereas the lowest (0.268) was between population 1 and population 3 (Table 5). All the pairwise *F*_*ST*_ values appeared to be significant (*p* < 0.05).

**Table 5:** Pairwise *F*_*ST*_ values (below diagonal) and geographic distance in kilometers (above diagonal) between studied *Cerastium alpinum* populations

	1	2	3	4	5	6	7
1	–	0.19	0.33	0.55	745.89	3254.63	2092.4
2	0.304	–	0.14	0.36	745.7	3254.67	2092.44
3	0.268	0.300	–	0.23	745.57	3254.65	2092.44
4	0.538	0.381	0.375	–	745.34	3254.74	2092.53
5	0.680	0.658	0.559	0.777	–	3535.48	2434.46
6	0.756	0.718	0.661	0.786	0.839	–	1166.2
7	0.657	0.625	0.537	0.753	0.773	0.831	–

The results of AMOVA showed that most of the identified genetic variation occurred among populations (61.69%), whereas the remaining 38.31% was attributed to variation within populations (Table 6).

**Table 6:** Partitioning of diversity found in *Cerastium alpinum* from all analyzed populations using AMOVA (*F*_*ST*_ = 0.6169)

Source of variation	Degrees of freedom	Sum of squares	Variance components	Percentage of variation
Among population	6	2265.891	19.421	61.69
Within population	126	1519.839	12.062	38.31
Total	132	3785.729	31.483	

Significance tests (1023 permutations); *p* = 0.001

**Fig. 3:**
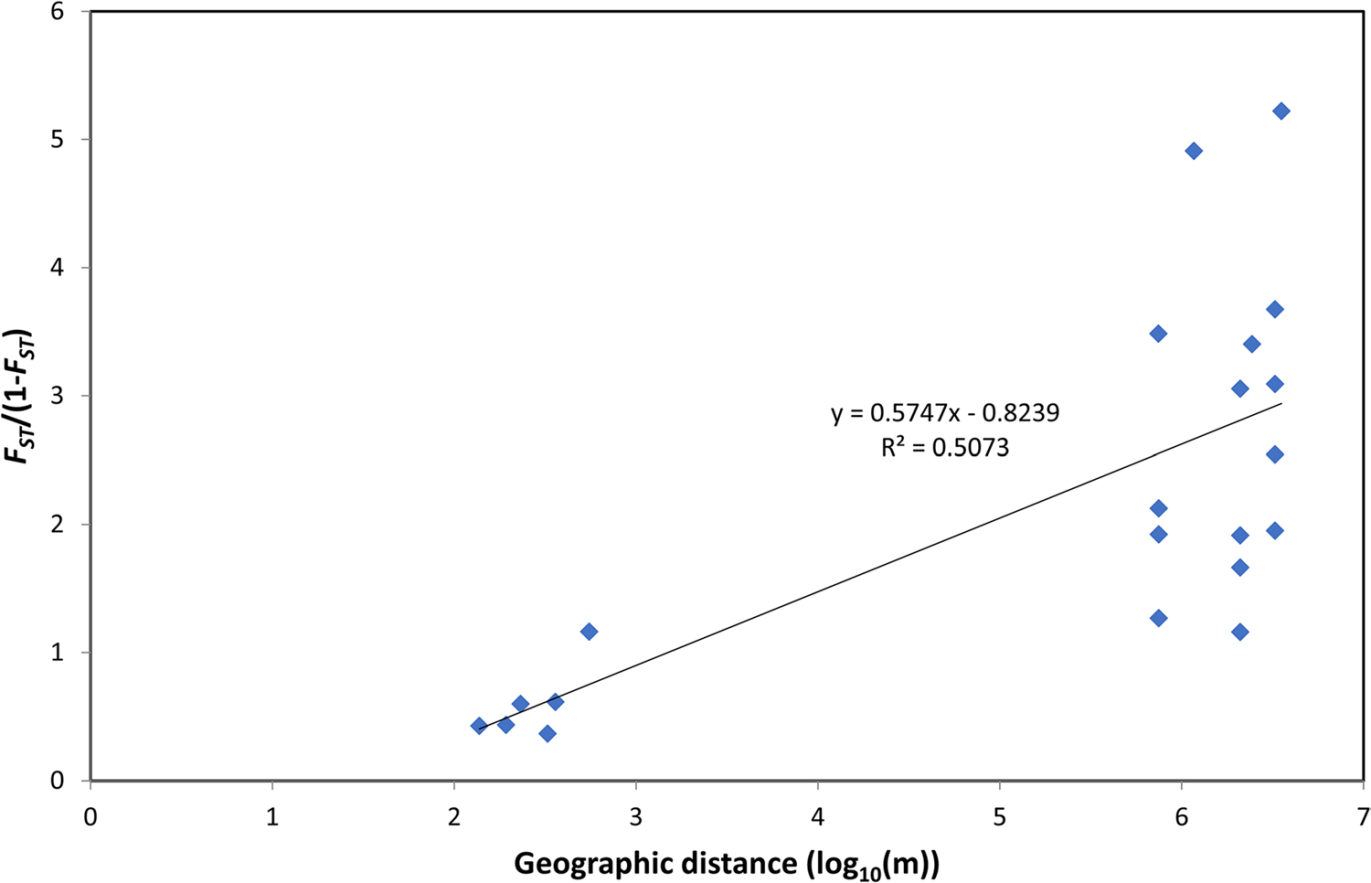
Plot of the isolation by distance (IBD) analysis. The Mantel test scatterplot shows the pairwise *F*_*ST*_/(1 − *F*_*ST*_) as a function of the logarithm of the geographical distances between studied populations of *Cerastium alpinum*

The IBD analysis revealed a significant correlation between genetic divergence and the logarithm of the geographic distance between populations (*R*^2^ = 0.5073, *p* = 0.02), which means that the genetic distance between populations, expressed as pairwise *F*_*ST*_/(1 − *F*_*ST*_) values, increases with the spatial distance between them (Fig. 3, Table 5).

### Neutrality tests and demography

Tajima’s *D* did not show any deviation from zero except for population 6, which was negative and significant. Fu’s *F*_*S*_ was negative for all populations (except population 6), but only for populations 5 and 7 did it significantly deviate from zero (Table 7). The *F*_*S*_ statistic interpretation was performed according to Fu (1997), who indicated that it should be considered significant at the 5% level if its *p* value is below 0.02 but not below 0.05. In the mismatch analysis for population growth and geographic expansion, SSD values were not statistically significant, and all samples had a low raggedness index (Table 8).

**Table 7:** Tajima’s *D* test and Fu’s *F*_*S*_ neutrality tests of seven populations of *Cerastium alpinum*

Test	Description	Populations							Statistics	
		1	2	3	4	5	6	7	Mean	SD
Tajima’s *D* test	*S*	68	74	120	58	35	67	43	66.429	27.501
	*P*_*i*_	22.506	33.341	42.942	21.967	12.046	11.735	14.353	22.698	11.740
	Tajima’s *D*	0.877	1.919	1.523	1.093	0.745	− 1.719	0.533	0.710	1.171
	Tajima’s *D p* value	0.854	0.989	0.952	0.900	0.809	0.031	0.757	0.756	0.330
Fu’s *F*_*S*_ test	Theta *p*_*i*_	22.506	33.341	42.942	21.967	12.046	11.735	14.353	22.698	11.740
	Exp. no. of alleles	16.101	11.838	21.756	12.233	11.315	10.811	11.490	13.649	3.983
	*F*_*S*_	− 3.047	− 0.703	− 5.073	− 4.488	− 6.382	0.290	− 7.103	− 3.787	2.786
	*F*_*S*_* p* value	0.125	0.309	0.033	0.023	0.007	0.564	0.003	0.152	0.212

*S*, number of segregating sites; *P*_*i*_, mean number of pairwise differences; Theta *p*_*i*_, Watterson’s theta based on *S*; *F*_*S*_, Fu’s *F*_*S*_; *SD*, standard deviation

**Table 8:** Estimates of mismatch analysis of seven populations of *Cerastium alpinum*

Model	Statistics	Populations							Mean	SD
		1	2	3	4	5	6	7		
Demographic expansion	SSD	0.014	0.046	0.009	0.022	0.008	0.035	0.010	0.021	0.015
	Model (SSD) *p* value	0.380	0.130	0.740	0.460	0.370	0.200	0.400	0.383	0.196
	Raggedness index	0.009	0.025	0.005	0.014	0.013	0.048	0.023	0.020	0.014
	Raggedness *p* value	0.790	0.620	0.890	0.890	0.590	0.370	0.270	0.631	0.245
Spatial expansion	SSD	0.014	0.038	0.011	0.023	0.008	0.034	0.010	0.020	0.012
	Model (SSD) *p* value	0.560	0.230	0.510	0.450	0.280	0.160	0.300	0.356	0.151
	Raggedness index	0.009	0.025	0.005	0.014	0.013	0.048	0.023	0.020	0.014
	Raggedness *p* value	0.910	0.910	1.000	0.990	0.600	0.470	0.230	0.730	0.300

*SD*, standard deviation; *SSD*, sum of squared deviations; *Raggedness index*, Harpending’s raggedness index

Three tests (SIGN, standardized differences, and Wilcoxon sign-rank) were used to analyze the bottleneck effect in studied populations (Table 9). In each case, the values were statistically significant, except for population 6 (SIGN test), population 4 (standardized differences test), and populations 4 and 6 (one-tailed Wilcoxon test for heterozygosity excess). Also, the results of all three tests showed that there were significant signs of bottleneck effects in populations 1, 2, 3, 5, and 7.

**Table 9:** Testing the bottleneck versus mutation-drift equilibrium hypotheses for all analyzed populations (IAM mutation model)

Population	SIGN test	Standardized test	Wilcoxon test		
			One tail for heterozygosity deficiency	One tail for heterozygosity excess	Two tails for heterozygosity excess and deficiency
**1**	Hee = 29.73 Hd = 22 He = 45 *p* = 0.00014	T2 = 5.423 *p* = 0.00000	1.00000	0.00000	0.00000
**2**	Hee = 33.37 Hd = 18 He = 55 *p* = 0.00000	T2 = 2.500 *p* = 0.00621	0.99739	0.00266	0.00531
**3**	Hee = 57.01 Hd = 28 He = 91 *p* = 0.00000	T2 = 7.053 *p* = 0.00000	1.00000	0.00000	0.00000
**4**	Hee = 27.72 Hd = 19 He = 38 *p* = 0.00457	T2 = 1.355 *p* = 0.08769	0.70143	0.30131	0.60262
**5**	Hee = 17.28 Hd = 6 He = 28 *p* = 0.00014	T2 = 3.897 *p* = 0.00005	0.99994	0.00007	0.00014
**6**	Hee = 32.89 Hd = 33 He = 33 *p* = 0.53768	T2 = − 2.838 *p* = 0.00227	0.00127	0.99876	0.00253
**7**	Hee = 20.94 Hd = 12 He = 30 *p* = 0.00376	T2 = 3.098 *p* = 0.00097	0.99087	0.00946	0.01892

*Hee*, expected heterozygosity excess; *Hd*, heterozygosity deficiency; *He*, heterozygosity excess

## Discussion

### Genetic diversity and differentiation of *Cerastium alpinum*

*Cerastium alpinum* occurs mainly in the Northern Hemisphere, North America (Canada and Greenland), Europe, and Western Siberia (Hultén 1956). Our research focused on the only natural Polish stand of *C. alpinum* (in the Babia Góra National Park) and populations of the species from Switzerland (Piz Val Gronda, Alps), Sweden (Nuolja massif, Abisko National Park), and Norway (Svalbard, Kaffiøyra).

*Cerastium alpinum* is a common perennial in Fennoscandia. In alpine regions, it has a continuous distribution on alpine heat and serpentine soils, while in boreal forests, it has a scattered distribution on ultramafic soils and steep slopes (Nyberg Berglund et al. 2001; Nyberg Berglund and Westerbergh 2001). Ultramafic soils (often called serpentine) are characterized by low concentrations of plant nutrients and high, potentially toxic, concentrations of Mg and Ni (Proctor 1999). Although Mg is one of the vital nutrients associated with chlorophyll health, the increased concentration of Mg creates conflict with other ions, like Ca, and may decrease their uptake (Nyberg Berglund et al. 2003). In the case of Ni, increased concentrations of these ions were reported to be responsible for the inhibition of cell division at root meristems (Robertson 1985). Moreover, nickel’s negative effect on photosynthesis and transpiration regulation was observed (Carlson et al. 1975). Research on *C. alpinum* showed that individuals growing in serpentine soils produce a lower number of seeds, display a more dwarfed structure, and show a higher tolerance to Ni and Mg stress (Grundt et al. 1999; Nyberg Berglund et al. 2003). In the Alps, the species has a scattered distribution and can be found on stony grasslands and ridges of the alpine and subalpine zones (The National Data and Information Center on the Swiss Flora, http://www.infoflora.ch). In Poland, *C. alpinum* occurs only in one place, on the Babia Góra massif in the Babia Góra National Park. This species is critically endangered in Poland due to its small range and geographical isolation (Parusel 2001).

Environmental and geographic isolation can strongly influence a population’s genetic structure and diversity (Wang and Bradburd 2014). This isolation can lead to decreased genetic variation and increased inter-population genetic differences due to reduced gene flow. That could be especially observed in alpine landscapes, which, due to their heterogeneous topography, may create numerous geographically and ecologically isolated habitats for alpine plants (Körner 2003). The terrain relief with steep valleys and high mountain ridges may limit gene flow and lead to more genetic differentiation between isolated plant populations compared to plants from less isolated habitats or lower altitudes (Till-Bottraud and Gaudeul 2002). Many factors, such as population size and the mating system, can affect genetic variation. Genetic diversity in small populations may also be limited by genetic drift and/or bottleneck during colonization by a small number of colonists and subsequent inbreeding (Ellstrand and Elam 1993; Lowe et al. 2004; Stöcklin et al. 2009). As a result, the lower ability of a population to respond to environmental changes might be observed, associated with the accumulation of deleterious recessive alleles and increased mortality of seeds and seedlings, which increases the probability of population extinction (Young et al. 1996; Lowe et al. 2005). High-mountain areas are also characterized by many stress factors, such as extreme temperatures, shorter vegetative periods, strong winds, heavy rainfall, and increased UV radiation, which affect plant growth and development (Körner 2003). Adaptive evolution driven by these factors favors individuals (genotypes) with traits that enhance survival and reproduction and consequently shapes the genetic diversity of populations (Bertel et al. 2018). The reproductive system also influences the genetic diversity of a plant population. Outcrossing species usually have higher within-population diversity and lower differentiation among populations than selfing species (Loveless and Hamrick 1984).

All the factors mentioned above also exist for *C. alpinum* on the Babia Góra massif. However, the unique character of *C. alpinum* from that location has not received adequate attention, which is reflected in a deficient number of published studies. The earliest scientific report describing the occurrence of *C. alpinum* on Babia Góra was released in 1906 by Zapałowicz (1906). Later reports included more detailed descriptions of the species’ morphology, occurrence, and abundance (Parusel 1993, 2001, 2008, 2014; Borysiak and Stachnowicz 2004, 2018). So far, no genetic studies have been performed on *C. alpinum* from Babia Góra, so we lack knowledge of its historical and current genetic composition. Genetic evaluation of these valuable genetic resources is essential not only to complement our state of knowledge but also for developing and managing effective conservation strategies for the species in Poland. Therefore, genetic characteristics of the *C. alpinum* from the Babia Góra massif seem to be a very urgent task, especially in light of the present and possible future threats, for example, intensive and increasing tourism in this area and the consequences of global climate change. The mountain environment is an ideal model for studying physiological and adaptive responses to global climate change (Körner 2003). In the next 50–80 years, the amount of rain and snow in Europe’s alpine areas is expected to go down, while the temperature is expected to go up (Engler et al. 2011). In high-elevation areas, the growing season could even get 60 days longer (Raible et al. 2006).

Due to progressive climate warming, plants found at high altitudes are particularly vulnerable to increasing competition due to the upward migration of species previously located at lower altitudes (Lenoir et al. 2008; Chen et al. 2011; Rumpf et al. 2018). Unfortunately, no such research has been carried out so far for the Babia Góra massif and its vicinity. However, the increased temperature and changes in precipitation appeared as the most likely drivers of changes in plant species composition observed within the last 90 years in the Tatra Mountains (Czortek et al. 2018), the mountain range located about 50 km southeast of the Babia Góra massif. Czortek et al. (2018) found that species composition changed the most in snowbeds, mylonite grasslands, and hygrophilous tall herb communities.

To the best of our knowledge, no investigations using iPBS markers for *C. alpinum* or any other member of the genus *Cerastium* have been carried out. Results of our study proved the suitability of this technique for evaluation of genetic diversity of the species. Two hundred and eight (79.39%) of the detected amplification products in our study on the genetic diversity of *C. alpinum* using the iPBS method were polymorphic, and the average polymorphism was found to be at 25.35% (across loci and populations). From 13.36% in population 5 to 45.80% in population 3, this parameter’s value was found. There were even higher levels of polymorphism in earlier studies using iPBS markers, such as 82.35% for *Nicotiana tabacum* (Yaldiz et al. 2018), 85.7% for *Psidium guajava* (Mehmood et al. 2013), 86.3% for *Vitis* varieties (Guo et al. 2014), 97.4% for *Myrica rubra* (Fang-Yong and Ji-Hong 2014), and 98.7% for Peruvian rosewood (Baloch et al. 2022). For Antarctic plants, however, a substantially lower level of polymorphism was noted, such as an average of 12.5% for *Deschampsia antarctica* (Androsiuk et al. 2020) and 9.57% for *Colobanthus quitensis* (Koc et al. 2018). Even lower variability between clones of the apricot cultivar was found by Baránek et al. (2012) at 4.88%. Twenty of the total 208 polymorphic bands were found to be private bands, meaning they only appeared in one population and were not present in the others. Populations 3 and 6 had the most private alleles (5 and 6, respectively), but population 1 only had one such amplification result. The only population for which none of these factors was scored was population 2. Unfortunately, these unique amplification products cannot be treated as diagnostic markers as they were not observed in all individuals representing particular populations.

Our study revealed low genetic diversity within populations of *C*. *alpinum* (mean *H*_*e*_ = 0.085). The highest value of that parameter was observed for population 3 (*H*_*e*_ = 0.162) and the lowest for population 5 (*H*_*e*_ = 0.051). Several authors found similarly low levels of heterozygosity in other isolated plant populations. The arctic-alpine species *Dryas octopetala* forms isolated populations in mountainous regions on northern Greece’s southernmost edge of the species’ distribution range. Studies by Varsamis et al. (2021) aimed at evaluating genetic variation within three selected populations of the species and analyzing its overall genetic structure. The results indicated relatively low intrapopulation genetic diversity (mean value of *H*_*e*_ = 0.156). In *Pilosella alpicola* subsp. *ullepitschii*, an endemic plant in the Carpathians, in the Western Carpathians (Slovakia and Poland), this species occurs at many sites, but only four sites are known in the Eastern and Southern Carpathians (Romania). Studies by Šingliarová et al. (2008) with the use of allozyme markers indicated a significant loss of genetic diversity (*H*_*e*_ = 0.134) in isolated populations of the Eastern and Southern Carpathians in comparison to populations from the Western part (*H*_*e*_ = 0.235). The results suggested that this population experienced a robust genetic bottleneck, probably due to a founder effect. On the other hand, studies of the influence of alpine habitat isolation on the genetic diversity of the rare and isolated population of *Campanula thyrsoides* showed a large genetic variation (*H*_*e*_ = 0.762; Ægisdóttir et al. 2009; *H*_*e*_ = 0.714; Frei et al. 2012) within the populations and a relatively low inbreeding coefficient. This rare monocarpic perennial’s high genetic diversity within its own population is best explained by the fact that its long-lived individuals do not breed with each other and that its generations overlap.

Significant traces of a recent reduction in effective population size were noted for all *C. alpinum* populations in this study. However, negative and significant Fu’s *F*_*S*_ statistics were observed only for populations 5 and 7, whereas negative and significant Tajima’s *D* was only for population 6, confirming a demographic expansion of the populations mentioned above. It might suggest that the studied *C. alpinum* populations might experience different demographic histories or extinction-recolonization events.

The results of our study revealed that despite a low level of polymorphism revealed by iPBS markers (on average, 25.35% of iPBS bands revealed differences between individuals from the particular population) and low genetic diversity (average *H*_*e*_ = 0.085), the analyzed populations of *C. alpinum* were characterized by very high genetic differentiation (*F*_*ST*_ = 0.6169, results of Structure software which assigned individuals into seven probable clusters, and high population dispersal revealed by PCoA). The significant pairwise *F*_*ST*_ values between all pairs of studied populations and the significant results of IBD analysis suggest that this population differentiation could be attributed to limited gene flow between them. This is, however, a surprising observation in the case of *C. alpinum* populations from the Babia Góra massif, which are not separated by high geographical distances (the pairwise distance between populations from this site ranges from 0.14 to 0.55 km). Therefore, the most likely explanation is the seed dispersal method (gravity) and the fact that the *C. alpinum* seeds are not morphologically adapted to wind dispersal.

Isoenzymatic analysis of *C. alpinum* from 31 populations from Fennoscandia also revealed their high genetic differentiation (*F*_*ST*_ = 0.526) (Nyberg Berglund et al. 2006). The authors also observed that some populations were fixed for some alleles and/or were characterized by inbreeding. The isolation of these populations was pointed out as the most likely explanation for the observed pattern of genetic variation (Nyberg Berglund et al. 2006).

### Implication for conservation

Genetic diversity is a fundamental source of biodiversity and can be defined as any measure that quantifies the magnitude of genetic variability within a population (Hughes et al. 2008). Therefore, evaluating genetic diversity and population differentiation is vital for species conservation (Carvalho et al. 2019). There are two complementary conservation strategies for plant species: in situ (on-site) conservation that protects species in their native habitat, while ex situ (off-site) protects endangered species outside their natural habitat in artificial environments, e.g., botanical gardens, arboreta, or as a germplasm collection in gene banks (Maunder et al. 2001; Schoen and Brown 2001; Heywood and Iriondo 2003; Guerrant et al. 2004; Havens et al. 2006; Hardwick et al. 2011; Miller et al. 2016). The general concept of in situ conservation is the conservation of living resources in the surroundings where they have developed and to maintain certain species in a dynamic relationship with the natural habitat (Edwards and Kelbessa 1999). This strategy enables the conservation of a large amount of natural genetic diversity. However, it requires adequate human and financial resources to ensure the effectiveness of protected areas in maintaining biodiversity (Zegeye 2017). However, because of climate change, possible pressure from invasive species, or habitat degradation, we cannot always completely prevent the extinction of a species in its natural environment. In that case, ex situ conservation seems to be the only alternative.

The popularity of seed banking is due to the ease of storage and immediate access for scientists, as well as resistance to habitat destruction, diseases, and predators (Roberts 1991; Schoen and Brown 2001). In addition, the deposited material can be used for species reintroduction to their natural habitat or as a genetic rescue, i.e., to increase the diversity of genetically depleted populations (Schoen and Brown 2001). However, despite the many advantages of ex situ conservation, it is not indifferent to the protected resources—it limits genetic variability and eliminates natural processes (e.g., evolutionary and ecological processes) that act on the particular species and are responsible for the adaptation of the species to changing environmental conditions. One example of ex situ conservation efforts that did not meet expectations is associated with *Cochlearia polonica* (Brassicaceae). It is an endemic plant species that is extinct in the wild in Poland and is known only from one transplanted population (by the Centuria River, right bank tributary of the Biała Przemsza river, in Southern Poland) with similar habitat conditions to its native site. Some individuals were transplanted to the Botanical Garden of the Polish Academy of Sciences in Warsaw to protect the species from complete extinction. After 18 years of ex situ conservation, Rucińska and Puchalski (2011) studied genetic diversity within the garden and source population. They observed a decrease in genetic diversity within the botanical garden population (Nei’s gene diversity = 0.1007) compared with the source population (0.1558). Similar results were observed for *Silene otites* (Lauterbach et al. 2012) and *Metasequoia glyptostroboides* (Li et al. 2005). So, in situ and ex situ conservation strategies should not be seen as alternatives, but rather as ways to protect endangered species that work well together. Both have their pros and cons, but both have been shown to be important and effective ways to do so.

Populations with more genetic diversity are more likely to be able to adapt to changing environmental conditions because they have more genetic variation (Willi et al., 2006). This makes them better candidates for conservation efforts. But there are also examples of both plant (e.g., *Arabidopsis lyrata*; Huber et al. 2014, Takou et al. 2021) and animal (e.g., *Oncorhynchus mykiss*; Willoughby et al. 2018, *Salvelinus fontinalis*; Yates et al. 2019) species that show that even populations with very little genetic diversity (e.g., because of a bottleneck) can have distinct signatures of local adaptation. Therefore, the low genetic diversity of *C. alpinum* populations from Babia Góra should not be treated as a direct threat to the population’s survival. What is more, *C. alpinum* from Babia Góra is characterized by higher genetic diversity than other studied populations of the species from Sweden, Switzerland, or Norway (Svalbard). Even lower values of *H*_*e*_ were observed with the application of the same DNA markers (iPBS) for populations of *C. quitensis* (*H*_*e*_ = 0.036; Koc et al. 2018), *D. antarctica* (*H*_*e*_ = 0.021; Androsiuk et al. 2020), and *Poa annua* (*H*_*e*_ = 0.063; Androsiuk et al. 2019). In all the above-mentioned cases, the populations’ adaptation to harsh climatic conditions of the Maritime Antarctic was indicated as the most probable explanation of observed patterns of genetic variation. This may also be true for *C. alpinum* populations whose genetic pools have been subjected to strong selection from climatic and edaphic factors found in mountainous habitats.

Considering the low genetic diversity and high population differentiation revealed in this study, we suggest that all *C. alpinum* populations from the Babia Góra massif should be treated the same during the development of its conservation strategy, as only then most of the genetic variability can be preserved. Furthermore, the results of botanical inventory studies of the Babia Góra massif clearly show that the area of occurrence of *C. alpinum* is shrinking (Perzanowska 2012), so there is an urgent need to develop an efficient strategy for species protection. Fortunately, all Polish *C. alpinum* sites are located within the protected area of Babia Góra National Park, which will help to maximize the effects of activities associated with species conservation. Nevertheless, due to the constant threat from tourism (trampling, unauthorized plant collection), regular monitoring of the species abundance and population condition should be continued.

## Conclusions

Currently, we observe the increasing recognition of the value of genetic data to support the decisions associated with developing and managing species conservation strategies. In this paper, the retrotransposon-based DNA markers (iPBS) proved helpful in revealing the genetic variation of *Cerastium alpinum* populations. The results showed a relatively low level of genetic diversity and high population differentiation. Furthermore, the genetic structure analyses reveal that the *C. alpinum population* from Babia Góra forms a distinct and heterogeneous group with greater genetic diversity than populations from other regions. Although *C. alpinum* populations from Babia Góra revealed significant traces of a recent bottleneck, like all other studied populations of the species, they still represent valuable genetic resources deserving protection. This has become especially important in light of the recent observations pointing to the shrinking area of its occurrence. Therefore, immediate action is required to develop an efficient conservation strategy to protect this species’s only Polish location.

## Supplementary Information

Below is the link to the electronic supplementary material.

Supplementary file1 (DOCX 16 KB)

Supplementary file2 (XLSX 117 KB)

Supplementary file3 (RAR 72.1 MB)
