# Supplementary material for: Genetic variation of Cerastium alpinum L. from Babia Góra, a critically endangered species in Poland
Source: J Appl Genet. 2022 Nov 2;64(1):37–53. doi: 10.1007/s13353-022-00731-x (PMC9837003; doi:10.1007/s13353-022-00731-x)
Supplement: Supplementary file 1 — Supplementary file1 (DOCX 16 KB) [file 13353_2022_731_MOESM1_ESM.docx]

**Table S1.** Evaluation of PCR efficiency for tested PBS primers.

| **Primer** | **Sequence** | **PCR efficiency ^a^** |
| --- | --- | --- |
| 2074 | GCTCTGATACCA | 1 |
| 2076 | GCTCCGATGCCA | 2 |
| 2079 | AGGTGGGCGCCA | 3 |
| 2080 | CAGACGGCGCCA | 1 |
| 2085 | ATGCCGATACCA | 3 |
| 2217 | ACTTGGATGTCGATACCA | **5** |
| 2220 | ACCTGGCTCATGATGCCA | 0 |
| 2221 | ACCTAGCTCACGATGCCA | 2 |
| 2224 | ATCCTGGCAATGGAACCA | 2 |
| 2228 | CATTGGCTCTTGATACCA | **4** |
| 2229 | CGACCTGTTCTGATACCA | **4** |
| 2231 | ACTTGGATGCTGATACCA | 2 |
| 2232 | AGAGAGGCTCGGATACCA | **4** |
| 2237 | CCCCTACCTGGCGTGCCA | 0 |
| 2238 | ACCTAGCTCATGATGCCA | 3 |
| 2240 | AACCTGGCTCAGATGCCA | 0 |
| 2241 | ACCTAGCTCATCATGCCA | 1 |
| 2242 | GCCCCATGGTGGGCGCAA | 2 |
| 2249 | AACCGACCTCTGATACCA | 3 |
| 2251 | GAACAGGCGATGATACCA | **4** |
| 2253 | TCGAGGCTCTAGATACCA | 3 |
| 2272 | GGCTCAGATGCCA | **5** |
| 2277 | GGCGATGATACCA | **5** |
| 2373 | GAACTTGCTCCGATGCCA | 2 |
| 2374 | CCCAGCAAACCA | 1 |
| 2376 | TAGATGGCACCA | 1 |
| 2378 | GGTCCTCATCCA | **5** |
| 2381 | GCAACGGCGCCA | 1 |
| 2389 | ACATCCTTCCCA | 1 |
| 2391 | ATCTGTCAGCCA | 2 |
| 2393 | TACGGTACGCCA | 2 |
| 2395 | TCCCCAGCGGAGTCGCCA | 1 |
| 2399 | AAACTGGCAACGGCGCCA | 1 |
| 2415 | CATCGTAGGTGGGCGCCA | 3 |

^a^ PCR efficiency rating scale according to Kalendar et al. (2010): 0 – no bands; 1 – few and weak bands; 2 – a few strong bands; 3 – ≈10 strong bands; 4 – many bands (good primer); 5 – many strong and equally amplifying bands
